# Macrophages as carriers of boron carbide nanoparticles dedicated to boron neutron capture therapy

**DOI:** 10.1186/s12951-024-02397-5

**Published:** 2024-04-15

**Authors:** Anna Wróblewska, Bożena Szermer-Olearnik, Agnieszka Szczygieł, Katarzyna Węgierek-Ciura, Jagoda Mierzejewska, Dawid Kozień, Paulina Żeliszewska, Roksana Kruszakin, Paweł Migdał, Zbigniew Pędzich, Elżbieta Pajtasz-Piasecka

**Affiliations:** 1 Hirszfeld Institute of Immunology and Experimental Therapy, Polish Academy of Sciences, Weigla 12, Wrocław, 53-114 Poland; 2 Faculty of Materials Science and Ceramics, Department of Ceramics and Refractory Materials, AGH University of Krakow, Krakow, Poland; 3 Jerzy Haber Institute of Catalysis and Surface Chemistry, Polish Academy of Sciences, Kracow, Poland

**Keywords:** Macrophages, Dendritic cells, Cellular carriers, Nanoparticles, Boron carbide, Boron neutron capture therapy

## Abstract

**Background:**

The use of cells as carriers for the delivery of nanoparticles is a promising approach in anticancer therapy, mainly due to their natural properties, such as biocompatibility and non-immunogenicity. Cellular carriers prevent the rapid degradation of nanoparticles, improve their distribution, reduce cytotoxicity and ensure selective delivery to the tumor microenvironment. Therefore, we propose the use of phagocytic cells as boron carbide nanoparticle carriers for boron delivery to the tumor microenvironment in boron neutron capture therapy.

**Results:**

Macrophages originating from cell lines and bone marrow showed a greater ability to interact with boron carbide (B_4_C) than dendritic cells, especially the preparation containing larger nanoparticles (B_4_C 2). Consequently, B_4_C 2 caused greater toxicity and induced the secretion of pro-inflammatory cytokines by these cells. However, migration assays demonstrated that macrophages loaded with B_4_C 1 migrated more efficiently than with B_4_C 2. Therefore, smaller nanoparticles (B_4_C 1) with lower toxicity but similar ability to activate macrophages proved to be more attractive.

**Conclusions:**

Macrophages could be promising cellular carriers for boron carbide nanoparticle delivery, especially B_4_C 1 to the tumor microenvironment and thus prospective use in boron neutron capture therapy.

**Graphical Abstract:**

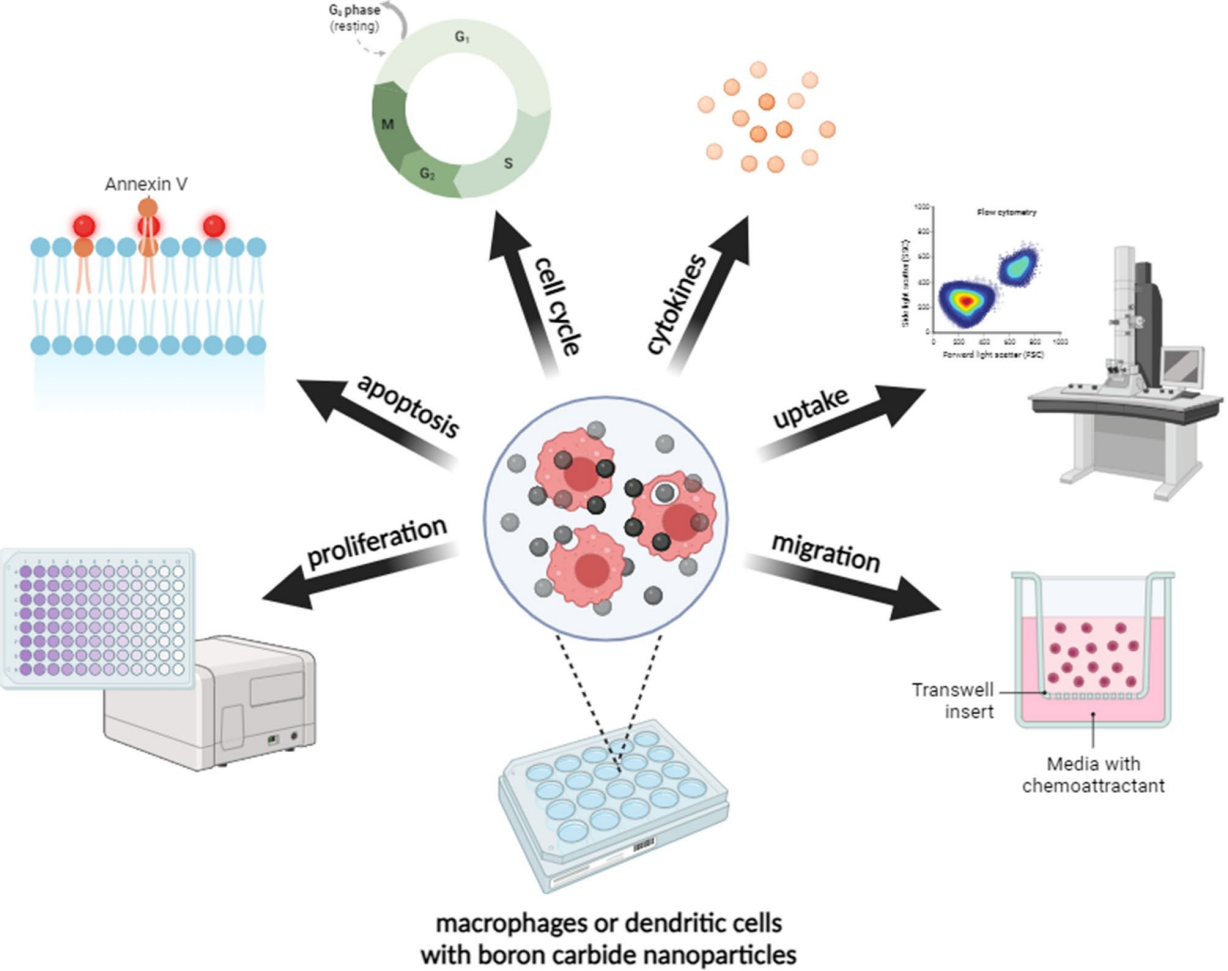

**Supplementary Information:**

The online version contains supplementary material available at 10.1186/s12951-024-02397-5.

## Background

Phagocytic cells are innate immune cells, including neutrophils, monocytes, macrophages, and dendritic cells. They play a crucial role in the immune response due to their ability to engulf pathogens and foreign particles, as well as present antigens and activate lymphocytes [1]. The use of phagocytic cells as carriers of nanoparticles is becoming a promising strategy in anticancer therapy called the “Trojan Horse”. Mainly due to their natural properties such as the ability to engulf foreign particles, non-immunogenicity, biocompatibility, degradability, a long half-life, the ability to cross biological barriers e.g. blood-brain barriers, tropism to hypoxia and accumulation in the tumor microenvironment. However, the most abundant population of tumor-infiltrating immune cells are macrophages, accounting for up to 50% of the tumor mass. Therefore, loading nanoparticles into macrophages can ensure their effective delivery to the tumor microenvironment by reducing their cytotoxicity, protecting them from rapid degradation and improving distribution in the body [2, 3]. In order to obtain efficient cellular carriers, nanoparticle characteristics such as size, shape, surface chemistry and mechanical properties, which have a significant impact on the kinetics of their internalization by phagocytic cells, should be taken into account [4, 5]. Importantly, the nanoparticles loaded in cellular carriers cannot be toxic to them and affect their proper functioning, as well as the cells must still be able to migrate toward the tumor [6].

The use of phagocytic cells as carriers of chemotherapeutic drugs is extensively studied. However, another promising application of these cells in anticancer therapy may be the delivery of boron-rich compounds to the tumor environment as a part of boron neutron capture therapy (BNCT), which may be a new type of radioimmunotherapy. BNCT is targeted radiotherapy intended for the treatment of patients with tumors located in hard-to-reach areas and do not respond to conventional treatment, such as gliomas, melanomas, head and neck cancers. BNCT is based on the selective delivery of compounds containing the non-radioactive boron-10 (^10^B) isotope to cancer cells, followed by irradiation of the tumor site with a beam of thermal or epithermal neutrons. Boron, due to its large cross-section, captures neutrons, resulting in the fission of its nucleus, followed by the release of recoiling lithium-7 (^7^Li) nuclei and alpha particles (^4^He) with a high linear energy transfer (LET), as well as gamma radiation. The range of the generated LET of alpha particles is limited by the length of their path in the tissue (5–9 μm). This limits tissue damage to areas where at least 20 µg/g of ^10^B per weight of tumor has accumulated, thus sparing adjacent normal cells [7, 8]. Although research on BNCT has been conducted since the 1950s, it is still at the clinical trial stage. Mainly due to a lack of compounds that selectively deliver boron to cancer cells and limited access to neutron sources [9, 10]. One of the promising compounds studied in recent years is boron carbide (B_4_C). Due to its properties, B_4_C is mainly applied as a ceramic material for the production of abrasion-resistant molds, low-wear bearings, nozzles and ballistic armor, as well as in the nuclear industry as a neutron absorber due to its high cross-section and self-repair ability [11]. B_4_C can be obtained by various methods of preparation, such as carbothermal and magnesiothermic reduction, gas phase reaction, powder sintering, or polymer pyrolysis. As a result boron carbide exhibits a wide range of stoichiometries and amorphous and polycrystalline structures, characterized by a high content of boron [12]. Therefore, there is growing interest in boron carbide as a boron-rich compound for use in BNCT. Its ability to destroy tumor cells after irradiation with a neutron beam was confirmed in mouse B16F10 melanoma cells in vitro [13], as well as inhibition of B16-OVA tumor growth in vivo [14]. In addition, functionalized boron carbide showed significant accumulation in tumor compared to other tissues in the SAS tongue squamous cell carcinoma model [15], HeLa cervical cancer [16] and EMT6 breast cancer [17].

In our work, we propose a therapeutic strategy based on the use of macrophages and dendritic cells as boron carbide carriers for delivery to the tumor environment and thus prospective application in boron neutron capture therapy. Our research assesses the ability of macrophages and dendritic cells originating from commercially available cell lines and bone marrow-derived macrophages to interact with boron carbide preparations with various physicochemical properties. For this purpose, we evaluated the effects of two boron carbide preparations on cell viability, apoptosis induction, changes in the cell cycle, and cytokine production. Additionally, we determined the ability of cells to interact and uptake boron carbide preparations, as well as migrate after loading with nanoparticles. The obtained research provides evidence that macrophages may be promising carriers of boron carbide nanoparticles and thus find future application in boron neutron capture therapy.

## Methods

### Compounds

Two boron carbide preparations were synthesized, differing in physicochemical properties such as nanoparticle size (32 ± 10 nm for preparation 1 - B_4_C 1, and 80 ± 30 nm for preparation 2 - B_4_C 2), zeta potential and hydrodynamic diameter, as already described in our previous work [18].

### Preparation and characterization of bone marrow-derived macrophages

To obtain bone marrow cells, femurs and tibias were harvested from healthy 8-10-week-old female C57BL/6 mice. Subsequently, the marrow cavities in cleaned bones were flushed with RPMI-1640 medium (Gibco) supplemented with 3% heat-inactivated fetal bovine serum (FBS; Biowest) and centrifuged for 7 min at 192 × g in 4 °C. The isolated bone marrow cells were cultured in RPMI-1640 medium with the addition of 10% FBS ( Sigma-Aldrich) and 50 ng/ml recombinant murine macrophage colony-stimulating factor (M-CSF; ImmunoTools) with the medium change every two days. Cells were cultured in this condition for 8 days to obtain unpolarized bone marrow-derived macrophages (M0 BMDM), while to obtain M1 and M2 macrophages, after 7 days of culture with M-CSF, 20 ng/ml interferon-γ (IFN-γ; ImmunoTools) and 100 ng/ml lipopolysaccharide (LPS, from E. coli O111:B4; Sigma Aldrich) to polarize into M1 and 20 ng/ml interleukin 4 (IL-4; ImmunoTools) to polarize into M2 were added for 24 h.

The phenotypic characterization was assessed after 8 days of bone marrow culture to determine the percentage of cells differentiated into macrophages and their activation state. For this purpose, BMDM were stained with anti-F4/80 Alexa Fluor 700, anti-CD11b PerCP-Cy5.5, anti-CD86 PE-Cy7, anti-CD206 APC (all from BioLegend) and anti-CD40 PE (Becton Dickinson). The analysis was performed using flow cytometer LSRFortessa with Diva Software (Becton Dickinson) and NovoExpress software 1.3.0 (ACEA Biosciences, Inc.).

#### Cell culture

RAW264.7 and J774A.1 cells of the mouse macrophages line obtained from American Type Culture Collection (ATCC; TIB-71™ and TIB-67™, respectively) were maintained in Dulbecco’s modified Eagle’s medium (DMEM; ATCC) containing 10% FBS, 100 U/ml penicillin and 100 mg/ml streptomycin (all from Sigma-Aldrich).

JAWS II cells of immature mouse dendritic cell line received from ATCC (CRL-11,904™) were cultured on a 1:1 mixture of MEM-α (Gibco) and RPMI-1640 (Gibco) supplemented with 10% FBS, 0.5 mM sodium pyruvate, 100 U/ml penicillin, 100 mg/ml streptomycin (all from Sigma-Aldrich) and 5 ng/ml recombinant murine granulocyte-macrophage colony-stimulating factor (GM-CSF; ImmunoTools).

All cell cultures were maintained in a NUAIRE CO_2_ incubator (37 °C, 5% CO_2_, 95% humidity) at the Hirszfeld Institute of Immunology and Experimental Therapy, Polish Academy of Sciences, Wrocław, Poland.

### MTT cell viability assay

The bone marrow-derived macrophages (M0, M1 and M2) were placed in 96-well plates at a density of 0.15 × 10^5^ cells/well. After 24 h, boron carbide preparations (B_4_C 1 and B_4_C 2) were added at concentrations in the range from 0.1 to 200 µg/ml and incubated for 72 h. Next, cells were incubated with MTT dye (3-(4,5-dimethylthiazol-2-yl)-2,5-diphenyltetrazolium bromide; 5 mg/ml)(Sigma-Aldrich) for 4 h and then lysed overnight in lysis buffer (N,N-dimethylmethanamide, sodium dodecyl sulfate, and water) at 37 °C. The absorbance of the solubilized crystal of formazan was measured at 570 nm using a Thermo Labsystems Multiskan RC microplate reader (Thermo Fisher Scientific Inc.) with Genesis Lite 3.05 Software (Thermo Life Sciences). The half maximal inhibitory concentration (IC_50_) value was calculated using GraphPad Prism 8 software (GraphPad Software). MTT cell viability assay for lines: RAW264.7, J774A.1 and JAWS II was performed and described in our previous work by Kozień et al. [18]. MTT assays were performed in two independent experiments, each in triplicate. The IC_50_ was calculated for each experiment individually and averaged on the graph for two independent experiments.

### Annexin V/ propidium iodide apoptosis assay

The RAW264.7 and J774A.1 cells (0.5 × 10^5^ cells/well for 24 h incubation and 0.25 × 10^5^ cells/well for 72 h in 24-well plates), JAWS II cells (1 × 10^5^ cells/well for 24 h incubation and 0.5 × 10^5^ cells/well for 72 h in 24-well plates) were incubated with boron carbide preparations at concentrations 10, 50, 100 and 200 µg/ml, and BMDM (1.2 × 10^5^ cells/well for both times) at concentrations 10, 50 and 100 µg/ml for 24 and 72 h. After this time, cells were harvested, suspended in a binding buffer and centrifuged (10 min, 100 × g, 4 °C). Next, cells were stained with Annexin V protein conjugated with APC fluorochrome (Becton Dickinson) for 15 min at room temperature. BMDM were additionally stained with anti-F4/80 BV421 (BioLegend) for 45 min at 4 °C. To assess the percentage of dead cells, 10 µg/ml propidium iodide (PI; Invitrogen) was added. The cells were analyzed using flow cytometer LSRFortessa with Diva Software (Becton Dickinson). Scheme of flow cytometry analysis was performed using NovoExpress software 1.3.0 (ACEA Biosciences, Inc.). Apoptosis assays were performed in two independent experiments, each in triplicate.

### BrdU cell cycle assay

The RAW264.7, J774A.1 and JAWS II cells (1 × 10^5^ cells/well in 24-well plates) were incubated with boron carbide preparations at concentrations 10, 50, 100 and 200 µg/ml, and BMDM (1.2 × 10^5^ cells/well) at concentrations 10, 50 and 100 µg/ml for 24 h. After this time, 32 µM bromodeoxyuridine (BrdU; Becton Dickinson) solution was added for 1 h, then collected and centrifuged for 10 min at 500 × g. The pellets were suspended in 70% ethanol and stored at -20 °C.

For BrdU staining, fixed cells were centrifuged (5 min, 500 × g, 4 °C) and incubated with 2 M HCl, 0.5% Triton X-100 for 30 min at room temperature. Next, the suspension was centrifuged (10 min, 500 × g, 4 °C), resuspended in 0.1 M Na Borate pH 8.5 and centrifuged again (10 min, 500 × g, 4 °C). Pellets were resuspended in PBS with 1% FBS (Biowest), 0.5% Tween20 (Sigma-Aldrich) and 20 µg/ml ribonuclease A (RNase; Sigma-Aldrich) and stained with anti-BrdU-FITC antibody (Becton Dickinson) for 30 min at room temperature. After this time, cells were centrifuged (10 min, 500 × g, 4 °C) and resuspended in PBS with 5 µg/ml propidium iodide (Invitrogen). Samples were analyzed using flow cytometer LSRFortessa with Diva Software (Becton Dickinson). The scheme of flow cytometry analysis was performed using NovoExpress software 1.3.0 (ACEA Biosciences, Inc.). Cell cycle assays were conducted in three repetitions.

#### Interaction of boron carbide preparations with phagocytic cells

Boron carbide preparations at a concentration of 100 µg/ml were incubated with the cultured RAW264.7, J774A.1, JAWS II cells and BMDM (0.1 × 10^6^ cells/well) in 24-well plates for 24 h. After this time, the changes in size and granularity of cells were analyzed in triplicate for each sample based on forward scatter (FSC) versus side scatter (SSC) using flow cytometer LSRFortessa with Diva Software (Becton Dickinson). Flow cytometric dot plots were prepared using the NovoExpress software 1.3.0 (ACEA Biosciences, Inc.).

### Transmission electron microscopy with EDS analysis

The RAW264.7, J774A.1, JAWS II cells and BMDM (0.1 × 10^6^ cells/well in 24-well plates) were incubated with boron carbide preparations at concentrations of 100 µg/ml for 24 h. Next, cells were harvested, fixed in 2.5% buffered glutaraldehyde at pH 7.2, and contrasted with 2% osmium tetroxide in the dark for 2 h. After this time, cells were washed with buffer and contrasted with 2% uranyl acetate for 12 h. The cell samples were then passed through an ascending alcohol series of 30%, 50%, 70%, 90%, 96% and 99.8%, and embedded in a medium-hard epoxy resin. After polymerization, ultra-thin sections were prepared on an ultramicrotome (Leica). Sections of 60 nm were prepared from the resin blocks and placed on copper grids (400 Mesh) with formvar film and carbon coating. Imaging was performed using a JEOL F-200 transmission electron microscope. The elemental composition analysis was also conducted by energy-dispersive X-ray spectroscopy (EDS) using a JEOL microscope. Elemental analyses were performed in the STEM mode of the microscope and spectra analysis was carried out in the Analysis program JED Series.

### Determination of cytokine production

Cytokine production by cells after 24 and 72 h of exposure to boron carbide preparations was assessed using commercially available ELISA kits (IL-1β – eBioscience; IL-6, TNF-α - Invitrogen; IL-10 – Becton Dickinson) in triplicate according to the manufacturer’s instructions. The values on the heat map were calculated according to the formula log_10_(concentration (pg/ml) + 1).

### Scratch assay

The RAW264.7 macrophages were plated at a density of 0.5 × 10^6^ cells/well in 6-well plates in the complete growth medium. After 24 h, boron carbide preparations were added for 24 h at a concentration of 100 µg/ml. Next, when 100% cell confluency was achieved, a gentle scratch was made with a 200 µl tip in the center of the cell monolayer in the wells. The floating cells were removed by washing with phosphate-buffered saline and then the complete medium was added. The macrophages were incubated at 37 °C and imaged over time for 24 h using an Olympus CKX53 light microscope. The experiment was performed in three repetitions, but the images show a selected part of the well for each sample at 0, 4 and 24 h.

### Transwell assay

The RAW264.7 macrophages were incubated with boron carbide preparations at a concentration of 100 µg/ml for 24 h. After this time, macrophages alone (0.4 × 10^5^ cells) and loaded with B_4_C nanoparticles were suspended in 150 µl of medium without FBS and placed in Transwell inserts with a pore size of 8 μm (Corning Costar). 500 µl RPMI-1640 with 5% FBS as a control medium and 72-hour supernatant from MC38 mouse colorectal cancer cells (1.5 × 10^5^ cells/well in 1 ml of RPMI-1640 with 5% FBS in a 24-well plate) were added to the lower chambers. After 16 h of incubation at 37 °C and 5% CO_2_, the upper part of the inserts was wiped using a cotton swab to remove cells that did not migrate. Then, the cells on the inserts were stained with the RAL 555 kit (RAL Diagnostics) according to the manufacturer’s instructions. Migrated cells were observed by an Olympus CKX53 light microscope and counted using ImageJ software from 7 images of the central part of each insert.

### Statistical analysis

All data were analyzed using the GraphPad Prism 8 software (GraphPad Software, Inc.). The type of statistical analysis used is described in the captions under the figures. All statistically significant differences are presented on the graphs; otherwise, the differences were not significant.

## Results

### Preparation and characterization of bone marrow-derived macrophages

**Fig. 1:**
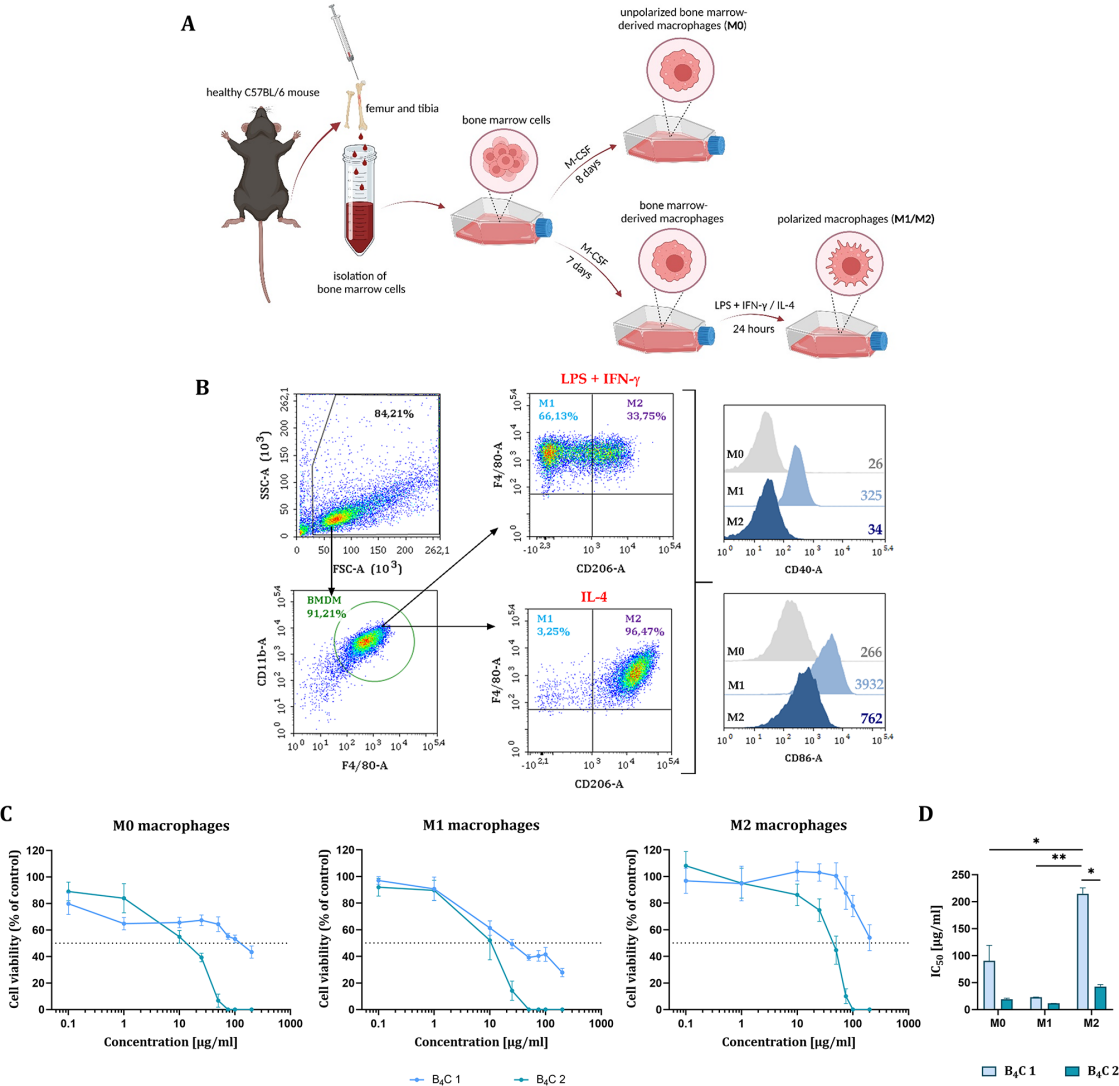
**A** Scheme of obtaining unpolarized (M0) bone marrow-derived macrophages (BMDM) and polarized to the M1 and M2 phenotypes. **B** Scheme of flow cytometry analysis showing phenotypic characterization of the BMDM obtained after 8 days of culture. Among the CD11b^+^F4/80^+^ population, M1 (CD206^−^) and M2 (CD206^+^) macrophages were identified in cell culture treated with LPS and IFN-γ, or with IL-4, respectively. Additionally, the expression of CD40 and CD86 molecules on the surface of BMDM was determined. **C** Effect of boron carbide preparations (B_4_C 1, B_4_C 2) on the viability of M0, M1 and M2 macrophages after 72 h of exposure determined by the MTT assay. The graphs represent the percentage of viable cells relative to control cells (Control = 100%). **D** Concentrations of B_4_C 1 and B_4_C 2 causing 50% inhibition of cell proliferation (IC_50_) calculated for each macrophage type. Results are expressed as means ± SD calculated for two independent experiments performed in triplicate. Differences between groups were calculated using two-way ANOVA followed by Tukey’s multiple comparison post-hoc test (*p < 0.05; **p < 0.01)

In our previous work, we performed a preliminary toxicity assessment of boron carbide preparations (B_4_C 1 and B_4_C 2) on cells originating from cell lines such as RAW264.7 and J774A.1 macrophages and JAWS II dendritic cells [18]. We decided to expand the research to include bone marrow-derived macrophages (BMDM), as it brings us closer to the in vivo model and provides a good basis for further experiments. For this purpose in the first step, bone marrow cells were isolated from the femurs and tibias of healthy mice and differentiated with M-CSF towards BMDM. The obtained macrophages were polarized with LPS and IFN- γ towards M1 macrophages or IL-4 towards M2 macrophages. Cells treated with only M-CSF were termed M0 macrophages (Fig. 1A).

BMDM were phenotypically characterized using flow cytometry as the CD11b^+^F4/80^+^ population. Macrophage polarity was determined based on CD206 expression, M1 macrophages were designated as CD206^−^ population and M2 as CD206^+^. In addition, the M1 phenotype was confirmed by the higher expression of CD40 and CD86 costimulatory molecules compared to M0 and M2 macrophages (Fig. 1B).

### Effect of boron carbide preparations on the cell viability

Our previous toxicity studies showed that RAW264.7 and J774A.1 macrophages were more sensitive to boron carbide preparations compared to JAWS II dendritic cells, especially to B_4_C 2 preparation containing larger nanoparticles [18]. Thus, similarly to determine the effect of boron carbide preparations on the viability of M0, M1 and M2 bone marrow-derived macrophages after 72 h of incubation, the MTT assay was performed (Fig. 1C). Based on the obtained results, B_4_C 1 and B_4_C 2 concentrations causing 50% inhibition of cell proliferation (IC_50_) for M0, M1 and M2 macrophages were calculated.

The results showed that all BMDM populations were more sensitive to B_4_C 2 than to B_4_C 1, but statistically significant sensitivity to B_4_C 1 depended on the type of macrophage subpopulation. The greatest inhibition of viability was observed for M1 macrophages after exposure to both B_4_C 1 (IC_50_ ∼ 23 µg/ml) and B_4_C 2 (IC_50_ ∼ 12 µg/ml) preparations. In contrast, the lowest toxicity was noticed for M2 macrophages after incubation with B_4_C 1 (IC_50_ ∼ 215 µg/ml) and B_4_C 2 (IC_50_ ∼ 43 µg/ml). Besides, differences in the sensitivity of these cells to B_4_C preparations compared to the other subpopulations proved to be statistically significant (Fig. 1D).

### Induction of apoptosis by boron carbide preparations

**Fig. 2:**
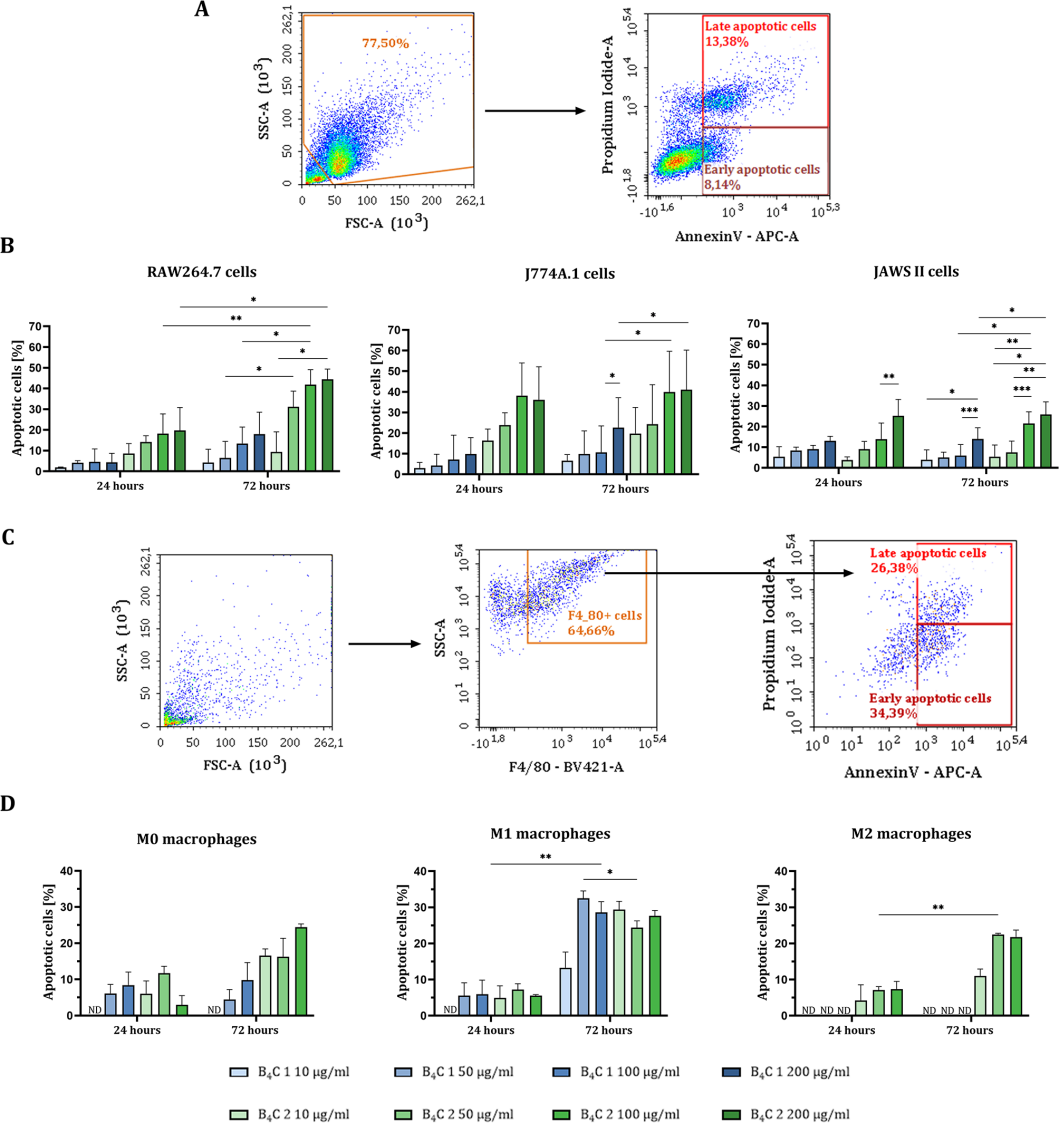
Evaluation of apoptosis induction in phagocytic cells by boron carbide preparations (B_4_C 1, B_4_C 2) using the Annexin V binding assay. **A** Scheme of flow cytometry analysis showing the method of determining cells from lines in early apoptosis (AnV^+^PI^−^) and late apoptosis (AnV^+^PI^+^) after exposure to boron carbide. **B** Percentage of apoptotic cells (early and late apoptotic cells) after 24 and 72 h of exposure to boron carbide preparations in RAW264.7, J774A.1 and JAWS II cells. **C** Scheme of flow cytometry analysis showing the method of determining F4/80^+^ bone marrow-derived macrophages in early apoptosis (AnV^+^PI^−^) and late apoptosis (AnV^+^PI^+^) after exposure to boron carbide. **D** Percentage of apoptotic cells (early and late apoptotic cells) after 24 and 72 h of exposure to boron carbide preparations in BMDM (M0, M1 and M2) populations. The graphs represent the percentage of apoptotic cells after deducting the percentage of apoptotic cells in the untreated control. ‘ND’ means no difference was detected between treated cells and untreated control. Results are expressed as mean + SD calculated for two independent experiments performed in triplicate. The differences between groups were calculated using the two-way ANOVA followed by Tukey’s multiple comparison post-hoc test (*p < 0.05; **p < 0.01; ***p < 0.001)

In order to determine the toxicity of boron carbide preparations after a short exposure time, the induction of apoptosis after 24 and 72 h of incubation with RAW264.7, J774A.1, JAWS II cells and BMDM was compared using the annexin V/propidium iodide assay. Early and late apoptotic cells were identified by flow cytometric analysis as annexin V positive and propidium iodide negative cells (AnV^+^PI^−^) and double positive (AnV^+^PI^+^), respectively (Fig. 2A). Early and late apoptotic cells were summed and presented as percentages of apoptotic cells in the graphs. The results showed an increase in the percentage of apoptotic cells in a concentration-dependent manner in RAW264.7, J774A.1 and JAWS II cells after boron carbide exposure. Additionally, in the case of RAW264.7 and J774A.1, the increase was also time-dependent. All phagocytic cells were more sensitive to B_4_C 2 than to B_4_C 1, especially macrophages (Fig. 2B). In the case of BMDM, apoptotic cells were identified among F4/80 positive cells due to the differentiation of macrophages from bone marrow cells and thus may not represent 100% of the obtained cell population (Fig. 2C). The percentage of apoptotic cells in M0 and M2 populations of BMDM increased in a time- and concentration-dependent manner, especially after exposure to B_4_C 2 preparation. While the percentage of apoptotic cells in M1 macrophages was time-dependent only. Similar to the viability assay, M1 macrophages were the most sensitive BMDM population and M2 the least sensitive to boron carbide preparations (Fig. 2D). Studies showed that the dependence of cell viability on the concentration of boron carbide and the exposure time of the preparation varies for cell types and their origin. Nevertheless, the B_4_C 2 preparation influenced the induction of apoptosis after 24 h in all tested cells. Therefore, to use higher concentrations of both preparations, further tests were carried out after 24 h.

### Effect of boron carbide preparations on the cell cycle arrest

**Fig. 3:**
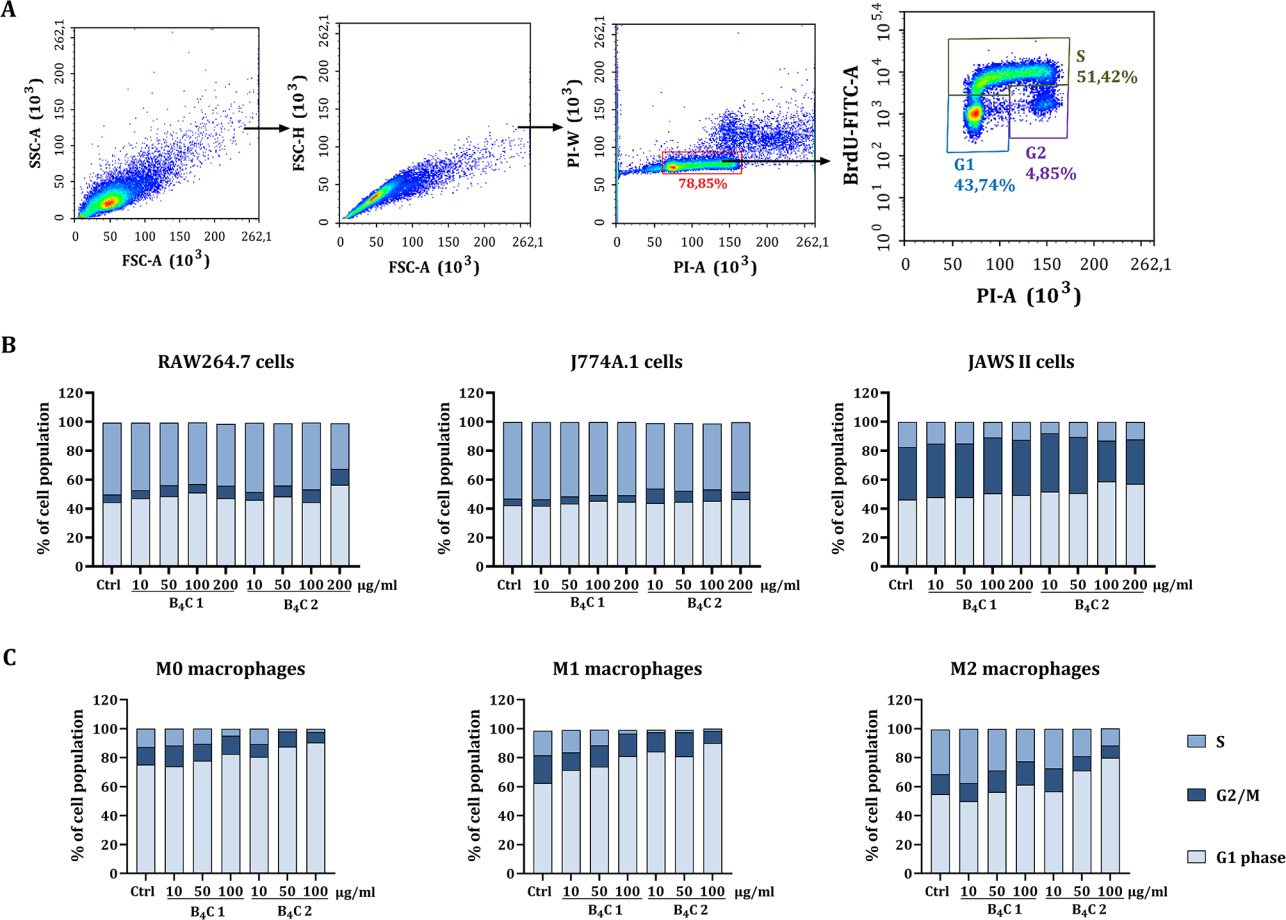
Assessment of the effect of boron carbide preparations (B_4_C 1 and B_4_C 2) on the cell cycle after 24-hour incubation with phagocytic cells using the BrdU assay. **A** Scheme of flow cytometry analysis showing the method of determining cell populations in S, G2/M and G1 phase. **B** Percentage of RAW264.7, J774A.1 and JAWS II cell population in S, G2/M and G1 phase after exposure to boron carbide at concentrations 10, 50, 100 and 200 µg/ml. **C** Percentage of M0, M1 and M2 macrophage population in S, G2/M and G1 phase after exposure to boron carbide at concentrations 10, 50 and 100 µg/ml. Results are expressed as means calculated for three repetitions

In addition, the BrdU assay was performed to analyze the effect of boron carbide preparations on potential changes in the cell cycle of RAW264.7, J774A.1, JAWS II cells and BMDM after 24 h of incubation. Cell populations in the G1, G2/M and S phases were identified by flow cytometry analysis (Fig. 3A). Boron carbide preparations did not significantly affect the cell cycle of RAW264.7, J774A.1, JAWS II cells (Fig. 3B). Incubation of M0, M1 and M2 bone marrow-derived macrophages with B_4_C preparations decreased the percentage of cells in the S phase in a concentration-dependent manner while increasing the percentage of cells in the G1 phase (Fig. 3C). However, the B_4_C 2 preparation showed a greater effect on reducing the population of BMDM in the S phase than B_4_C 1. B_4_C 2 at concentrations of 50 and 100 µg/ml reduced the population of M0 and M1 macrophages in the S phase to 1–2%. and at the same time induced the G1-phase arrest, which consequently gives cells the opportunity to undergo repair mechanisms or follow the apoptosis pathway. According to previous tests, M2 macrophages were the least sensitive from all BMDM subpopulations to boron carbide preparations.

### Interaction and uptake of boron carbide preparations by phagocytic cells

**Fig. 4:**
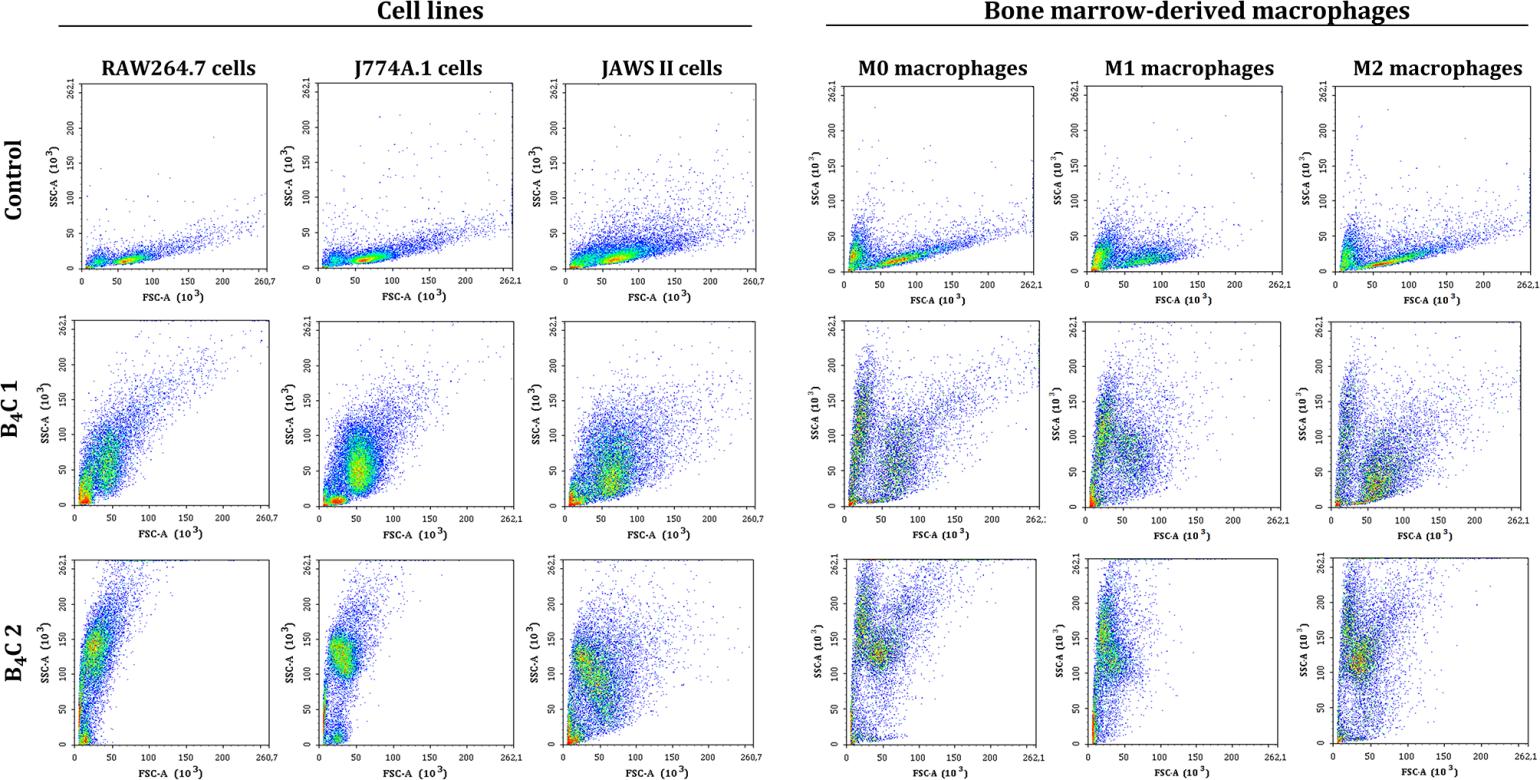
Flow cytometry dot plots demonstrating changes in cell size and granularity based on forward scatter (FSC) versus side scatter (SSC) for RAW264.7, J774A.1, JAWS II cells, and M0, M1, M2 bone marrow-derived macrophages after 24-hour exposure to boron carbide preparations (B_4_C 1 and B_4_C 2) at a concentration of 100 µg/ml compared to control untreated cells

**Fig. 5:**
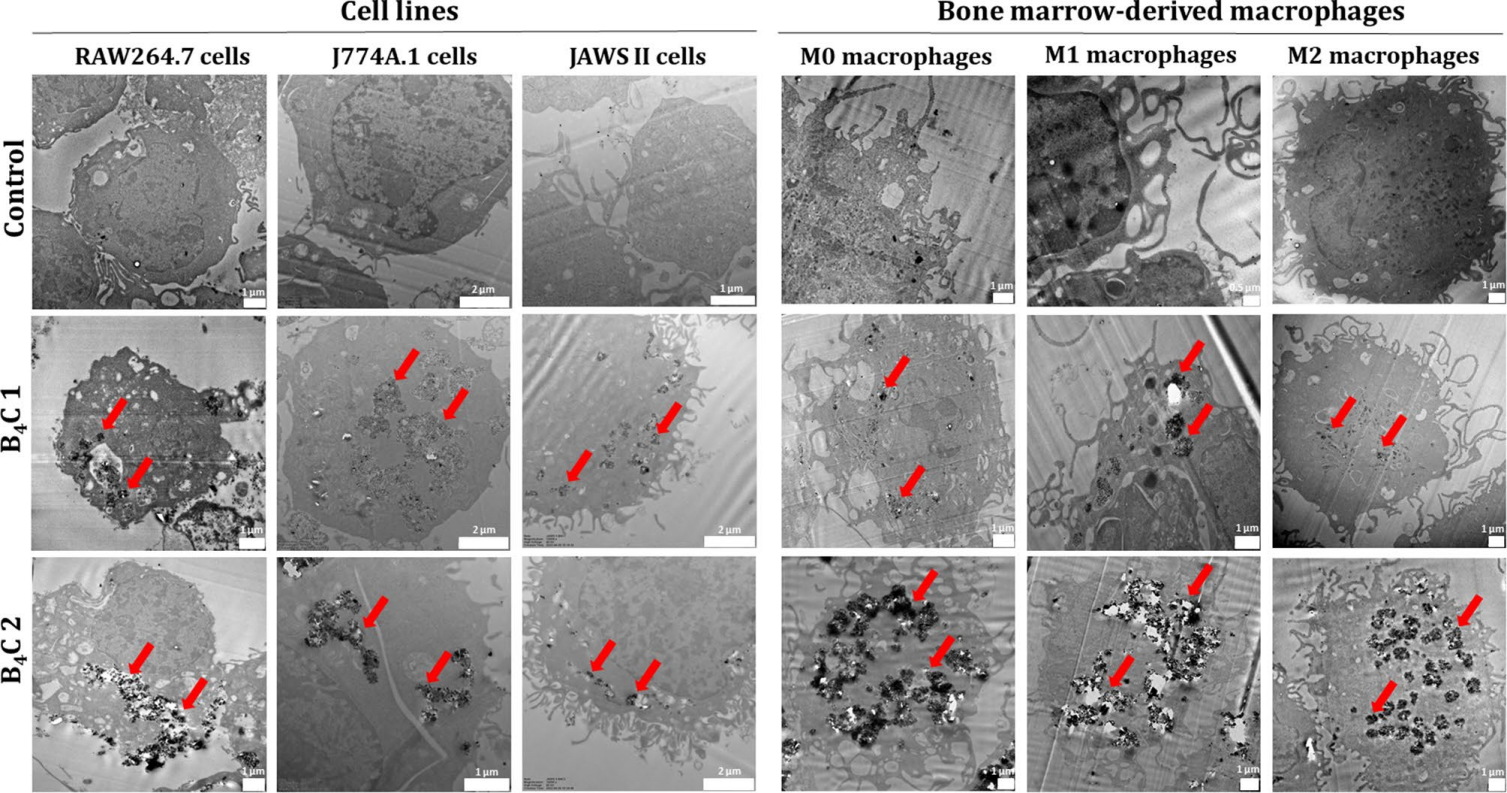
TEM images of ultra-thin sections of RAW264.7, J774A.1, JAWS II cells, and M0, M1, M2 bone marrow-derived macrophages after 24-hour exposure to boron carbide preparations (B_4_C 1 and B_4_C 2) at a concentration of 100 µg/ml compared to control untreated cells. Red arrows indicate exemplary locations of accumulated boron carbide in the cells

An important issue in the next step was to visualize the ability of phagocytic cells to interact with boron carbide preparations. For this purpose, after 24-hour incubation of boron carbide preparations with RAW264.7, J774A.1, JAWS II cells and bone marrow-derived macrophages, changes in cell size, and granularity were analyzed by flow cytometry based on forward scatter (FSC) versus side scatter (SSC). A more effective boron carbide interaction was observed for RAW264.7 and J774A.1 macrophages as well as BMDM compared to JAWS II dendritic cells (Fig. 4). The results suggest that an increase in cell granularity after incubation with boron carbide preparations, especially for B_4_C 2 containing larger nanoparticles could be the reason for both types of changes in the level of death and cell cycles of macrophages. These observations were confirmed by the transmission electron microscopy (TEM) technique, where ultra-thin cell sections obtained after 24-hour incubation with boron carbide were analyzed. In all cell samples, B_4_C 2 preparation was more visible than B_4_C 1, possibly due to the larger size of the nanoparticles and greater accumulation in the cells. Importantly, the highest accumulation was observed in M0, M1 and M2 bone marrow-derived macrophages and the smallest in JAWS II dendritic cells (Fig. 5). In addition, to identify and confirm the presence of boron in the cells, the elemental composition was analyzed by energy-dispersive X-ray spectroscopy (EDS) of RAW264.7, J774A.1 and JAWS II cell samples incubated with boron carbide preparations (Fig. S1).

### Cytokine production profile after exposure to boron carbide preparations

**Fig. 6:**
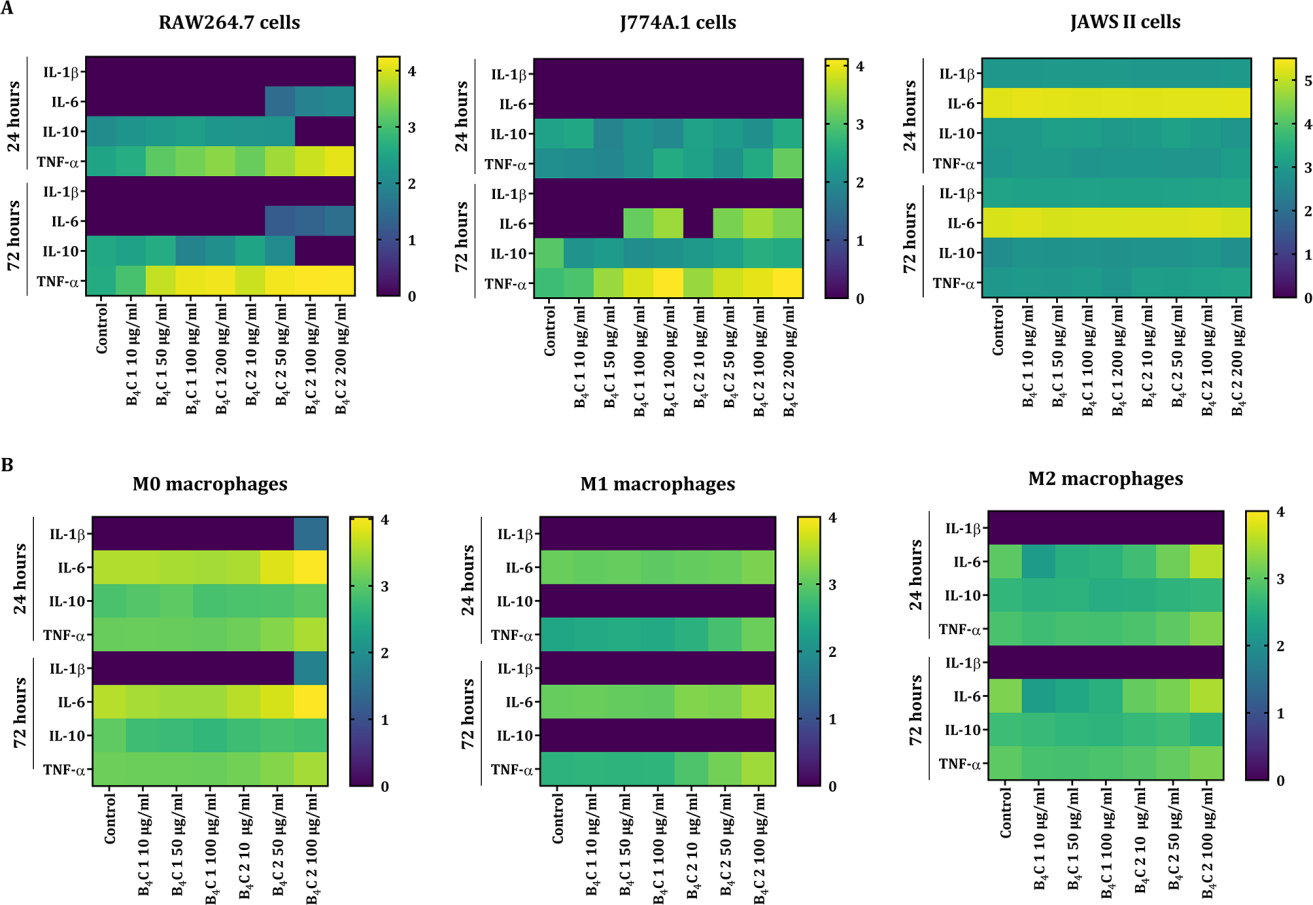
Heat map of cytokine production: IL-1β, IL-6, IL-10 and TNF-α by **A** RAW264.7, J774A.1, JAWS II cells and **B** M0, M1, M2 bone marrow-derived macrophages after 24 and 72 h of incubation with boron carbide preparations (B_4_C 1 and B_4_C 2). The values on the heat map were calculated according to the formula log_10_(concentration (pg/ml) + 1) from three repetitions

In order to investigate the effect of boron carbide preparations on the cytokine production profile in RAW264.7, J774A.1, JAWS II cells and BMDM, ELISA test was performed after 24 and 72 h of incubation. The production of anti-inflammatory cytokine IL-10 and pro-inflammatory cytokines such as IL-1β, IL-6 and TNF-α were assessed. An increase in TNF-α production in the concentration- and time-dependent manner was observed for RAW264.7 and J774A.1 macrophages (Fig. 6A), while in the case of BMDM populations only high concentrations of B_4_C 2 increased TNF-α production (Fig. 6B). An increase in IL-6 production by all types of macrophages was observed, especially after exposure to high concentrations of B_4_C 2 preparation. Boron carbide preparations did not significantly affect the production of IL-10 by these cells, only in the case of RAW264.7 cells, the high concentrations of B_4_C 2 inhibited the production of this cytokine. Furthermore, various ability to produce IL-10 was noted among BMDM, and it should be highlighted that M1 subpopulation cells did not release this cytokine compared to M0 and M2 macrophages. IL-1β was produced mainly by JAWS II dendritic cells, only a concentration of 100 µg/ml B_4_C 2 induced the production of this cytokine by M0 macrophages. Overall, JAWS II cells produced all analyzed cytokines at a constant level, regardless of the concentration of boron carbide.

### Migration ability of macrophages loaded with boron carbide nanoparticles

**Fig. 7:**
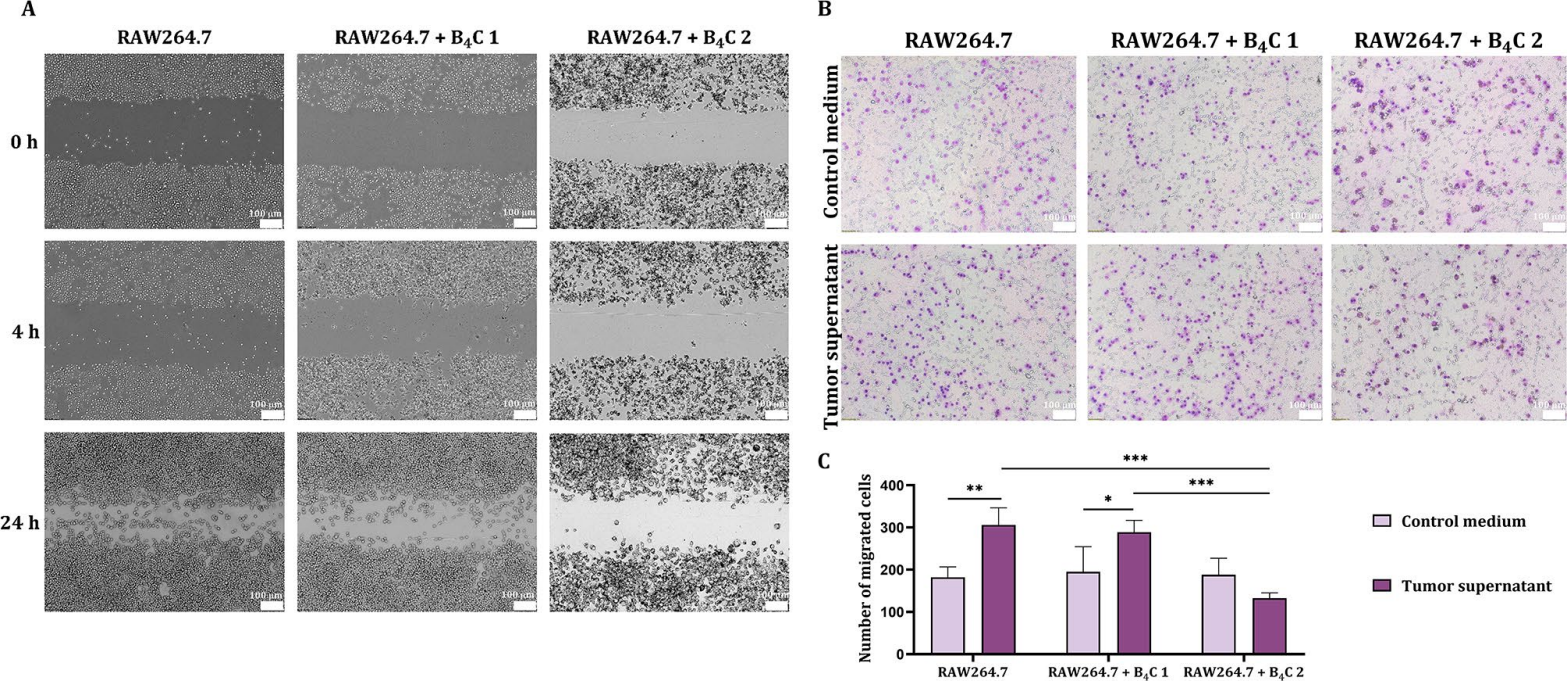
Assessment of the migration ability of RAW264.7 macrophages alone and loaded with B_4_C 1 and B_4_C 2 nanoparticles based on **A** Scratch assay and **B** Transwell assay into control medium and 72-hour supernatant from MC38 mouse colorectal cancer cells. **C** Comparison of the average number of migrated cells counted from 7 images of the central part of each insert. Differences between groups were calculated using two-way ANOVA followed by Sidak’s multiple comparison post-hoc test (*p < 0.05; **p < 0.01; ***p < 0.001)

In order to initially assess the functionality of using macrophages to transport boron carbide nanoparticles, the migration ability was assessed on RAW264.7 cells using Scratch and Transwell tests. The Scratch assay showed that B_4_C 1-loaded macrophages migrated in a similar manner to untreated cells after 24 h. While loading with B_4_C 2 nanoparticles significantly slowed down the RAW264.7 cell migration (Fig. 7A). Additionally, these observations were confirmed in the Transwell assay, in which B_4_C 1-loaded cells migrated more efficiently into the supernatant of MC38 colorectal cancer cells than into a control medium similar to untreated macrophages. The opposite effect was observed in the case of cells loaded with B_4_C 2, where the number of migrated cells was lower compared to the control macrophages (Fig. 7B,C). These results suggest that loading macrophages with B_4_C 2 nanoparticles disrupts their natural migration abilities.

## Discussion

The use of a cell-based delivery system in anticancer therapy is becoming a promising strategy. The greatest progress has been achieved in research on red blood cells, T lymphocytes, NK cells and mesenchymal stem cells, whose safety and effectiveness are being tested in clinical trials. However, macrophages and dendritic cells deserve special attention, primarily due to their natural ability to engulf foreign particles. An additional advantage of such carriers is specific tissue tropism, flexibility and low cytotoxicity [19, 20]. Sources of cellular carriers can be not only circulatory cells but also bone marrow-derived macrophages, peritoneal and alveolar macrophages, as well as dendritic cells derived from bone marrow or blood [6]. Therefore, our research were conducted on mouse macrophages from the RAW264.7 and J774A.1 cell lines and JAWS II dendritic cells, as well as bone marrow-derived macrophages in various states of polarization. RAW264.7 macrophages are a widely described model for loading with cytostatics, such as doxorubicin. Numerous studies confirmed that these macrophages can effectively uptake doxorubicin, migrate to the tumor microenvironment and release active drug in the tumor site. In the U87MG glioblastoma model, a high accumulation of doxorubicin-loaded RAW264.7 macrophages was observed [21], as well as tumor suppression, life extension and metastasis inhibition in 4T1 breast tumor-bearing mice [22]. In addition, RAW264.7 macrophages loaded with liposomes with encapsulated doxorubicin and gold nanorods were used for synergistic photothermal chemotherapy reducing tumor growth in 4T1-bearing mice [23].

In our work, we propose an innovative, previously undescribed strategy for using phagocytic cells as carriers of boron-rich compounds. This strategy can contribute to obtaining cellular boron carriers which, due to their natural properties, will selectively and efficiently deliver boron compounds into the tumor environment, achieving the desired therapeutic effect in boron neutron capture therapy. However, when developing such cellular carriers, it is crucial to select the appropriate concentration of nanoparticles that will not affect cell viability and their proper functioning, as well as the ability of cells to migrate, which will ensure the delivery and release of the nanoparticles in the tumor site. Therefore, in our study, we first assessed the effect of two boron carbide preparations on the viability, induction of apoptosis and cell cycle of phagocytic cells from cell lines and bone marrow-derived macrophages. We observed that the sensitivity of cells to boron carbide depends on the physicochemical properties of nanoparticles, the nature of the cells and their polarization state. Phagocytic cells from established lines were less sensitive than bone marrow-derived macrophages, which was particularly confirmed by cell cycle changes (Fig. 3). Therefore, the appropriate concentration of boron carbide nanoparticles for delivery via phagocytic cells in further studies must be individually selected depending on the cell types and should be below the IC_50_ value. In addition to the non-toxicity of boron carbide towards cellular carriers, the lack of systemic toxicity is also very important for boron compounds used in BNCT. Therefore, some studies report the effect of boron carbide on normal cells, such as human embryonic kidney cells (HEK-293), in which B_4_C nanoparticles were non-toxic in the tested concentration range up to 1200 µg/ml for 24 h of incubation [24]. While Türkez et al. confirmed that a 72-hour exposure to human lung alveolar epithelial cells inhibited cell viability by more than 50% at higher concentrations above 640 µg/ml [12]. In our studies, the inhibition of cell viability after 72-hour exposure to B_4_C 2 was observable at low concentrations, which is due to the natural ability of phagocytic cells to uptake and accumulate nanoparticles. In contrast, B_4_C 1 containing smaller nanoparticles was toxic only at high concentrations above 100 µg/ml. After comparing the induction of apoptosis by the two preparations of boron carbide in phagocytic cells, we observed that after 24 h in both all cell lines and BMDM, the percentage of apoptotic cells was lower than after 72 h (Fig. 2). These results suggest that to obtain effective cellular carriers, not only the selection of concentrations but also exposure time is crucial. Therefore in our previous studies, the uptake of boron carbide nanoparticles by RAW264.7 macrophages was confirmed by flow cytometry and fluorescence microscopy after 4 and 24 h of exposure [25]. Herein, we additionally observed an increase in cell granularity based on SSC in a flow cytometer (Fig. 4) and accumulated boron carbide in cells on TEM images (Fig. 5) after 24-hour exposure. Furthermore, EDS analysis was performed to confirm the presence of boron inside the cells, clearly indicating that the detected boron comes from nanoparticles (Fig. S1). Overall, B_4_C uptake was greater for macrophages from cell lines and BMDM than dendritic cells, which correlates with the results of cytotoxicity assays. In addition, greater changes in granularity and accumulation in TEM images were noted for the B_4_C 2 preparation compared to B_4_C 1. This phenomenon can be associated with the larger size of nanoparticles in B_4_C 2, because as is well known, the kinetics of compound internalization by phagocytic cells depends on their parameters such as size, shape, surface chemical composition and mechanical properties [4]. In our previous work, we described in detail the synthesis process of both boron carbide preparations and performed physicochemical characteristics, showing that both preparations differ, among others, in the size of nanoparticles, zeta potential, and hydrodynamic diameter [18]. Our results correspond with research that confirmed an increase in the uptake of gold nanoparticles by J774A.1 macrophages in a size-dependent manner [26], similarly, in the case of gliadin nanoparticles [27].

Internalization of nanoparticles by macrophages can affect changes in their phenotype, but the direction of these changes depends on the complex physicochemical properties of nanoparticles. It should be highlighted, that the phenotypic plasticity of macrophages is associated with the acquisition of appropriate biological functions in response to environmental stimuli, through changes in surface markers, gene expression profile, production of cytokines and chemokines, as well as metabolic properties [28]. In our studies, we observed a concentration- and time-dependent effect of boron carbide on the increase in the production of pro-inflammatory cytokines such as IL-6 and TNF-α by macrophages. However, significant changes were noted after exposure to B_4_C 2 containing larger nanoparticles. In contrast, both B_4_C preparations did not affect cytokine production by JAWS II dendritic cells (Fig. 6). Our results correspond to those obtained by Nishanth et al. who showed an increase in the production of IL-6 and TNF-α after exposure of RAW254.7 macrophages to carbon-coated silver, carbon black, and aluminum nanoparticles, but the greatest increase was noted after treatment with silver nanoparticles [29]. Research by Saborano et al. showed a significant release of TNF-α by RAW264.7 cells after exposure to high concentrations of silica nanoparticles [30]. Similarly, Park et al. confirmed that amorphous silica nanoparticles increased mRNA expression of pro-inflammatory genes TNF-α and IL-6 by peritoneal macrophages harvested from the treated mice [31]. In studies of bone marrow-derived macrophages, increased IL-6 and TNF-α release and gene transcription were also observed in a size- and concentration-dependent manner of iron oxide nanoparticles after 24-hour exposure [32].

Additionally, high concentrations of the B_4_C 2 preparation containing larger nanoparticles not only influenced the viability of phagocytic cells and their activation but also the migration of RAW264.7 macrophages towards the tumor environment. While cells loaded with the B_4_C 1 containing smaller nanoparticles migrated as well as the untreated control. Similarly, in the study by Zhang et al. RAW264.7 cells loaded with doxorubicin-silica nanocomplexes migrated the same as untreated macrophages [21]. Whereas Chang et al. showed that carboxylate-modified polystyrene latex beads of smaller size (30 and 50 nm) inhibited the migratory abilities of RAW264.7 macrophages. While loading with larger particles (500 nm) significantly increased the number of migrating cells [33]. These results confirm that the selection of the appropriate nanoparticle size is crucial for their effective uptake by cells and migration towards the tumor site.

In summary, macrophages showed a greater ability to uptake boron carbide nanoparticles than dendritic cells. Moreover, uptake of B_4_C 2 nanoparticles by macrophages was associated with significant toxicity and an increase in the production of pro-inflammatory cytokines as well as impaired the ability of these cells to migrate. Thus, B_4_C 1 is more promising for further research not only due to its lower toxicity but also its uptake by macrophages and ability to migrate towards the tumor environment.

## Conclusions

In conclusion, the development of cellular carriers for selective and effective nanoparticle delivery to tumor environment is a challenging and complex process that requires the precise refinement of conditions, such as the selection of the appropriate cell type, compound concentration and exposure time. Our research proved that macrophages, due to their natural abilities, could be promising carriers of boron carbide nanoparticles. Bone marrow-derived macrophages as primary cells are particularly attractive because they provide a more accurate model of in vivo macrophage physiology than those originating from cell lines. Moreover, loading these macrophages with smaller boron carbide nanoparticles (B_4_C 1) can ensure a negligible effect on viability and migration, thus effectively delivering B_4_C to the tumor environment in future in vivo studies. This research highlights the potential of using macrophages as boron carbide carriers in boron neutron capture therapy as a promising therapeutic anticancer strategy that can become a new type of radioimmunotherapy.

### Electronic supplementary material

Below is the link to the electronic supplementary material.

Supplementary Material 1

## Data Availability

The datasets used and analyzed during the current study are available from the corresponding author on reasonable request.

## Abbreviations

B_4_C: Boron carbide
BNCT: Boron neutron capture therapy
^10^B: Boron-10 isotope
^7^Li: Lithium-7 isotope
^4^He: Helium-4 isotope - alpha particles
LET: High linear energy transfer
FBS: Fetal bovine serum
M-CSF: Macrophage colony-stimulating factor
BMDM: Bone marrow-derived macrophages
IFN-γ: Interferon-γ
LPS: Lipopolysaccharide
IL: Interleukin
DMEM: Dulbecco’s modified Eagle’s medium
GM-CSF: Granulocyte-macrophage colony-stimulating factor
MTT: 3-(4,5-dimethylthiazol-2-yl)-2,5-diphenyltetrazolium bromide
IC_50_: Half maximal inhibitory concentration
TEM: Transmission electron microscopy
EDS: Energy-dispersive X-ray spectroscopy
TNF-α: Tumor necrosis factor α
