# Supplementary material for: Macrophages as carriers of boron carbide nanoparticles dedicated to boron neutron capture therapy
Source: J Nanobiotechnology. 2024 Apr 15;22:183. doi: 10.1186/s12951-024-02397-5 (PMC11017526; doi:10.1186/s12951-024-02397-5)
Supplement: Supplementary file 1 — Supplementary Material 1 [file 12951_2024_2397_MOESM1_ESM.docx]

**Supplementary Figures**


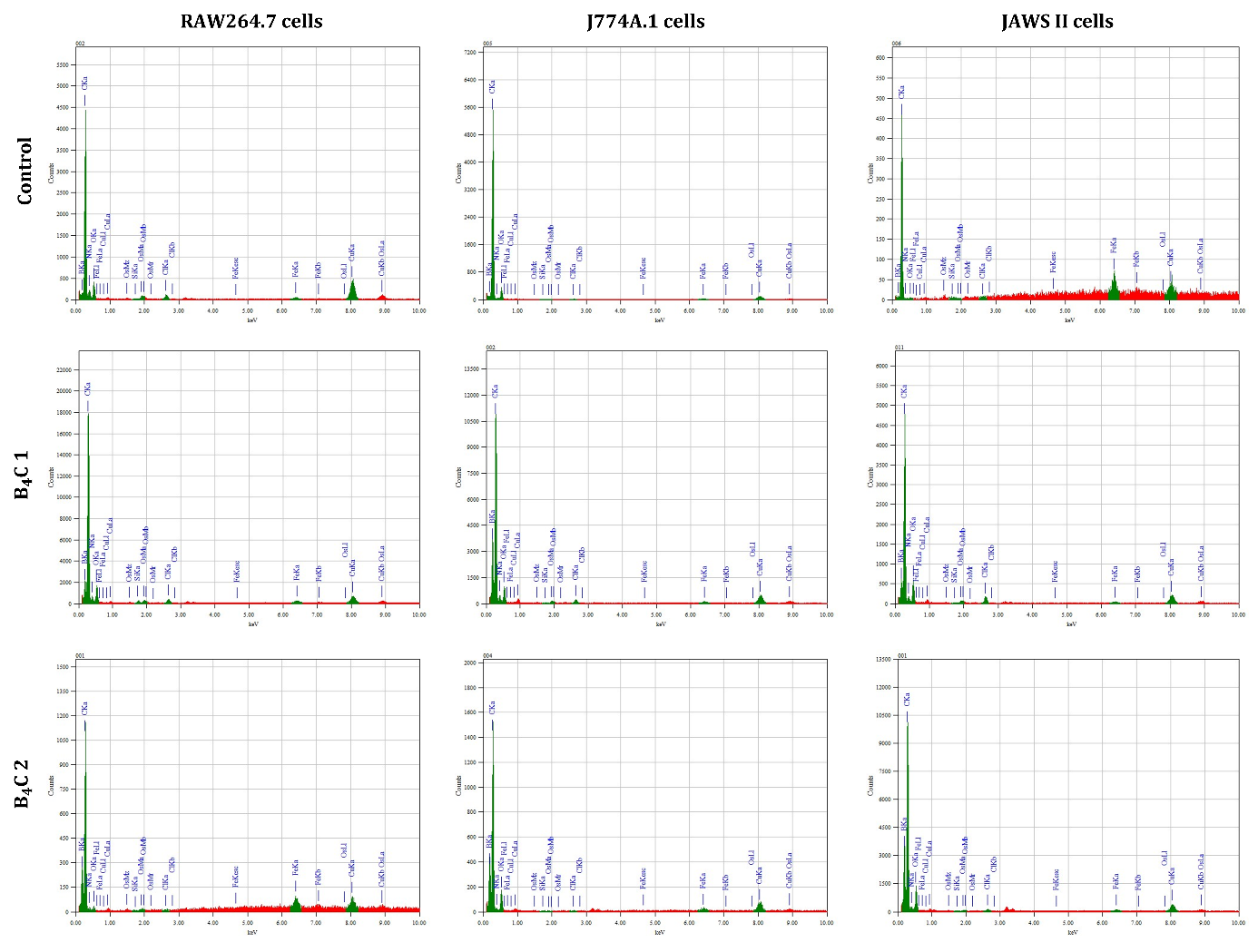


**Fig. S1** Energy dispersive X-ray spectroscopy (EDS) spectrum of the elemental composition of RAW264.7, J774A.1 and JAWS II cell samples treated with boron carbide preparations (B_4_C 1 and B_4_C 2) at a concentration of 100 µg/ml compared to control untreated cells.
